# Spontaneous Rupture of Aorta.—Hypodermic Use of Morphia

**Published:** 1879-04

**Authors:** H. L. Harrington


### Clinical Reports.

ARTICLE V.

NOTES FROM PRIVATE PRACTICE.

*I. Spontaneous Rupture of Aorta.*

S. S., female, aged 30, having been treated three days for acute dyspepsia by means of anodynes and laxatives, was on the evening of February 27, suddenly seized with most intense pain in the back; collapse immediately supervened, and death occurred in about one hour.

An autopsy nine hours later revealed a rupture of the abdominal aorta just above its bifurcation, the rent being about two centimeters in length; a large quantity (*fully two litres*) of coagulated blood was found behind the peritoneum; arterial coats discolored and softened from a point just above the origin of the renal arteries to the bifurcation; no aneurismal enlargement—indeed, the caliber of the vessel was less than usual; thoracic and abdominal organs nearly normal.

This case illustrates the necessity of autopsies in many instances; the friends of this patient firmly believed that she died of inflammation of the stomach, until they witnessed the post mortem, notwithstanding my assertion to the contrary.

*II. Hypodermic Use of Morphia.*

Was called a short time since to treat W. S., male, aged 62, for acute dyspepsia (bilious attack), accompanied by very severe pain. Administered hypodermically, in hypogastric region, morphia sulph. 0.02. Before the syringe was emptied, alarming syncope supervened, and recurred twice, at intervals of ten or fifteen minutes; stimulants administered freely, artificial respiration and the use of electricity were successful in reviving the patient. Neither narcotism nor coma were in any degree present. Is it possible to attribute the syncope to the effects of the drug? Not over fifteen seconds were occupied in the operation.

H. L. HARRINGTON.

